# Properties of Enhanced Calcium-alginate Beads as a Formulation for Disseminating the Entomopathogenic Nematodes *Heterorhabditis bacteriophora*, *Steinernema carpocapase*, and *Steinernema feltiae*

**DOI:** 10.2478/jofnem-2025-0020

**Published:** 2025-06-04

**Authors:** Reyhaneh Darsouei, Javad Karimi, Lukasz L. Stelinski

**Affiliations:** Department of Plant Protection, School of Agriculture, Ferdowsi University of Mashhad, Mashhad, Iran.; Department of Entomology and Nematology, Citrus Research and Education Center, University of Florida, Lake Alfred, USA.

**Keywords:** Biological control, infective juvenile, formulation, microbial control

## Abstract

Calcium alginate beads are one of the substrates used to formulate and store the infective juveniles (IJs) of entomopathogenic nematodes (EPNs). Due to the sensitivity of EPNs to heat, cooling devices are needed to transfer them from the production site to the field or greenhouse. Therefore, it is important to develop a formulation that can be stored at room temperature. The hypothesis of this study was that nematodes formulated in alginate beads would be more stable at room temperature than in sterile water. To compare and select the optimal conditions, formulations were tested at two temperatures: 25 and 8 Celsius (ºC). The formulation included sodium alginate (1.5–1.75%), glycerin, nematode suspension in distilled water (~15,000 IJs), food coloring, and two proprietary water-absorbent compounds. The suspension was added to CaCl_2_ (8:2 CaCl_2_: glycerin) to create calcium alginate beads. The beads were stored at 8 ºC and 25ºC with a control treatment consisted of sterile water. The study measured survival, reproduction rate, and infectivity of *Heterorhabditis bacteriophora, Steinernema carpocapsae*, and *S. feltiae* IJs in calcium alginate beads over time (1–180 days post-formulation). The survival rate of IJs in bead formulations was significantly higher than in the water control treatment. *H. bacteriophora* experienced higher survival at 25ºC, while *S. carpocapsae* experienced higher survival at 8ºC. *S. feltiae’s* survival was not affected by temperature. The pathogenicity of EPNs did not decrease over time. Calcium alginate effectively encapsulated living IJs of various EPN species, keeping them alive for up to three months at room temperature. These results suggest that alginate beads are a suitable material for EPNs formulation. Further research is needed to enhance the efficacy and economic viability of these formulations.

## Graphical abstract2

**Figure:**
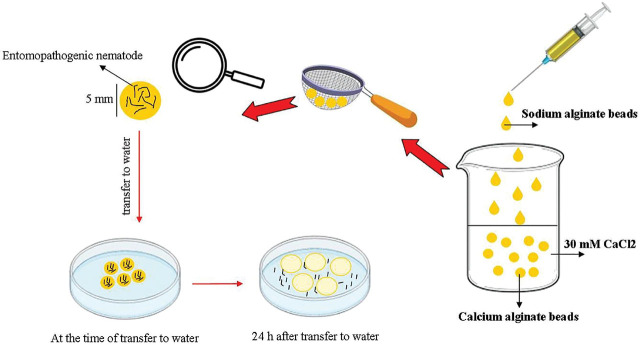

## 1. Introduction

Entomopathogenic nematodes (EPNs) from the families *Steinernematidae* and *Heterorhabditidae* are insect-parasitic organisms widely utilized as bioinsecticides (Glazer, 1992; Grewal, 2002; Karimi et al, 2018; Okram et al., 2025). They infect a wide range of insect hosts. Application of EPNs for control of soil-borne pests is effective because nematodes are protected against ultraviolet (UV) radiation (Kaya, 1990); However, environmental stress to EPNs applied against foliar pests remains a constraint (Glazer, 1992). EPNs have apparent potential for application as biopesticides, but their low survival in vitro at room temperature further constrains their practical use (Grewal, 2002). The third larval stage of EPNs, the infective juvenile (IJ) stage, is formulated as a product for field application. The IJs are free-living and searching for their eventual host, which is killed after infection. EPNs are suspended in the water and sprayed onto the surface of plants or soil. Commercial successes of EPN application are rare because IJs are very susceptible to UV (Gaugler et al., 1997) and spraying them onto the surface of plants results in high EPN mortality and/or loss (Shapiro-Ilan et al., 2006).

Given the constraints related application of EPNs as biopesticides in the field, recent studies have focused on improving formulations and application methods (Georgis and Kaya, 1998; Shapiro-Ilan et al., 2006; Aquino-Bolaños et al., 2019; Platt et al., 2019; Nxitywa and Malan, 2021; Fallet et al., 2024; Waweru et al., 2025). Suspensions of nematodes must be stirred continuously to achieve uniform mixtures. Additionally, IJs require high oxygen levels and are sensitive to elevated temperatures (Nxitywa and Malan, 2021). Moreover, formulations stored at room temperature are highly desirable, as this facilitates the transfer and application of nematodes in the field (Grzywacz et al., 2014; Ruiz-Vega et al., 2018). Nematodes should be formulated immediately after production, as dead nematodes can interfere with storage (Ehlers, 2007).

Recent research has focused on development of low-cost formulation capable of maintaining nematode viability at ambient temperatures (Ruiz-Vega et al., 2018). For long-term storage and transport, EPNs can be formulated using various carrier materials. Several studies have investigated the impact of different substrates on the longevity and pathogenicity of EPNs. Among these, sponge and vermiculite-based formulations are commonly employed for storing small quantities of nematodes. However, sponge formulations are not suitable for high nematode densities because IJs may migrate out of the sponge matrix. Additionally, IJs remain metabolically active within the sponge, leading to the depletion of stored energy reserves such as carbohydrates (Grewal, 2002; Grewal and Peters, 2005). A primary objective of EPN formulations is to reduce IJ activity and conserve their energy during storage. Both sponge and vermiculite formulations typically require refrigeration during storage and transportation to maintain nematode viability (Grewal, 2002). Alternative formulation approaches include the use of polyacrylamide gels, wettable powders, water-dispersible granules, alginate beads, clay, and activated charcoal (Bedding, 1988; Grewal, 2002; Chen and Glazer, 2005; Nxitywa and Malan, 2021).

Silver et al. (1995) developed granules composed of diatomaceous earth, hydroxyethyl cellulose, amorphous silica, fumed hydrophobic silica, lignosulfonate, starch, pregelatinized starch, and pre-gelled attapulgite clay, achieving a nematode survival rate of 90% after six weeks of storage at 25°C. Leite et al. (2018) evaluated the survival of *Steinernema feltiae* formulated in polyacrylamide gel and vermiculite at 25°C and 35°C, reporting higher survival rates of IJs in these substrates compared to water. Similarly, Grewal (2000) demonstrated that formulating IJs in water-dispersible granules reduces desiccation and enhances survival for up to three months at ambient temperature, outperforming aqueous formulations. Navon et al. (2002) encapsulated *S. carpocapsae* in an edible gel suitable for insect ingestion, achieving over 95% mortality of *Helicoverpa armigera* Hübner and *Spodoptera littoralis* Boisduval. Matadamas-Ortiz et al. (2014) encapsulated *S. glaseri* in diatomaceous earth pellets, reporting a 56% survival rate after 14 days at room temperature. Cruz-Martínez et al. (2017) observed that EPNs formulated and applied as infected insect cadavers exhibited higher pest control efficacy than those applied in aqueous suspension.

Formulations that suppress the metabolic activity of IJs enhance nematode survival. In certain formulations, IJs experience a reduction in water content, which leads to decreased activity (Kondo and Ishibashi, 1989). Alginate bead formulations have demonstrated the ability to limit nematode mobility (Georgis, 1990; Kaya and Nelson, 1985; Kaya et al., 1987). Alginate, a polysaccharide derived from brown algae (Yabur et al., 2007), was first utilized by Kaya and Nelson (1985) to encapsulate *Heterorhabditis heliothidis* and *Steinernema feltiae*. In this formulation, EPNs became immobile within the beads but were able to escape once the beads softened after several days—an attribute considered unfavorable for commercial applications (Hiltpold et al., 2012; Kim et al., 2015). To address this limitation, Kim et al. (2021) enhanced the calcium alginate formulation by incorporating 18% glycerol, which effectively induced a quiescent state in EPNs.

Calcium alginate beads are water-insoluble. The aim of this study was to develop a formulation that is water-soluble and stable at room temperature while also preserving the pathogenicity of nematodes over time. In this research, we investigated the potential for creating calcium alginate beads that could dissolve in water. Subsequently we assessed the survival and infectivity of IJs of three species of nematodes, *Heterorhabditis bacteriophora*, *Steinernema carpocapsae*, and *Steinernema feltiae* encapsulated within these beads and in sterile water. Our hypothesis was that nematodes enclosed in alginate beads would exhibit higher survival rates compared to those in sterile water. Additionally, we examined the survival of nematodes to address the issue of transferring nematodes to a cooling device. Furthermore, we analyzed the impact of varying sodium alginate concentrations on bead hardness and the release rate of IJs. Lastly, we investigated the pathogenicity and reproduction rate of nematodes throughout the formulation period, the pathogenicity and reproductive rate of IJs suspended within the formulated alginate beads, recognizing the significance of maintaining these traits throughout the formulation period.

## 2. Materials and methods

### 2.1. Insect and nematode rearing

*Tenebrio molitor* Linnaeus, 1758 (Coleoptera: Tenebrionidae) larvae were reared on wheat bran at 28°C and 60–65% RH (Shah et al., 2024). Three species of nematodes, *Heterorhabditis bacteriophora* Poinar 1976, *Steinernema carpocapsae* Weiser 1955, and *Steinernema feltiae* Filipjev 1934, were cultured in last-instar larvae of *T. molitor* at room temperature. The concentration used was 35 IJs/larvae. Dead larvae were transferred into White traps (White, 1927) until IJs emerged from cadavers.

### 2.2. Calcium alginate beads

To form calcium alginate beads, various concentrations of sodium alginate (0.5, 1, 1.5, 1.75, and 2%) and calcium chloride (CaCl_2_) (10, 15, 20, 30, 50, and 100 mM) were examined. Also, glycerin was used at various ratios in the calcium chloride suspension (0:10, 1:9, 2:8, 3:7, 4:6, and 5:5 glycerin).

### 2.3. Calcium alginate bead formation

**Figure 1:**
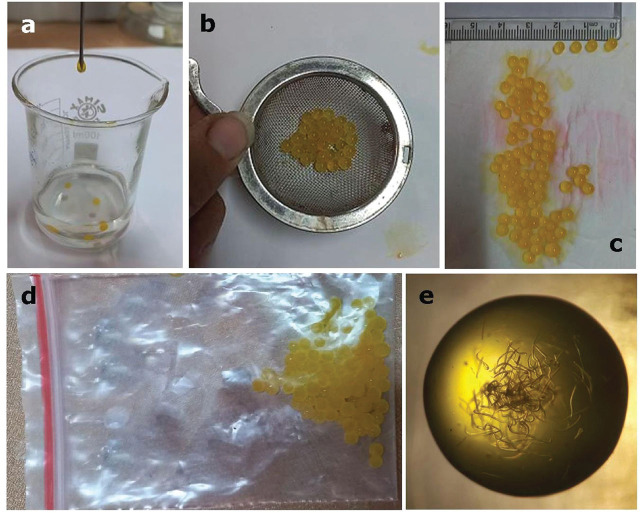
Formation of calcium alginate beads: A) Suspension added to 30 mM of calcium chloride; B) Suspension passed through strainer; C) Bead transferred to filter paper; D) Beads maintained in plastic zip cap; and E) IJs within calcium alginate beads.

Two concentrations (1.5 and 1.75 %) of sodium alginate were selected. The formulation was prepared in a total volume of 3.050 cc, consisting of 2 ml sodium alginate, 0.2 ml glycerin, 0.8 ml nematode suspension (~15,000 nematodes), and 50 μl of food coloring dye. The prepared suspension was introduced into a 30 mM CaCl2 solution (8:2 CaCl2) using an 18-gauge syringe (Shenzhou company; China). Two proprietary (patent pending) water-absorbent compounds were added to the suspension to enhance formulation performance. The mixture was shaken for 1 minute. The beads were then removed from the calcium chloride solution using a strainer and spread onto filter paper to remove excess water. The beads were stored in a plastic zip-lock bag (5×10 cm). The prepared formulation was kept at 8ºC and 25ºC for future experiments (Fig. 1). In the control treatment, IJs were kept in sterile water.

### 2.4. Nematode survival within beads over time

A factorial design was employed, with 1) rearing temperature and 2) sodium alginate concentration as the main effects. Ten beads were transferred into four ml of sterile water per treatment. The enumeration of IJs released from bead formulations was conducted using a stereomicroscope after a 24-hour incubation period. Living IJs were distinguished from dead individuals by their motility, whereas dead IJs were characterized by the presence of shriveled body surfaces or gas bubbles within their bodies (Peters, 2016). The survival rate was calculated as the proportion of living IJs to the total number of juveniles (both alive and dead) and expressed as a percentage. Observations were recorded at 10-day intervals over a period of 180 days following the encapsulation of EPNs of each species within bead formulations. The entire experiment was replicated twice.

### 2.5. Pathogenicity of IJs encapsulated within beads

The pathogenicity of the IJs escaping from the beads was quantified using *T. molitor* larvae using the abovementioned treatments. For the adverse control treatment, IJs were kept in sterile water. For each replicate, 10 final instar larvae of *T. molitor* were transferred into a Petri dish (8 cm diameter), and 35 IJs were added per larvae. For each treatment, 4 Petri dishes were used. Mortality of *T. molitor* larvae was recorded 48 hours after inoculation. Dead larvae were placed in White traps until IJs emerged from cadavers. Observations were made at 1, 30, 60, 90, 120, 150, and 180 days after preparing IJ treatments (formulations). The entire experiment was repeated twice.

### 2.6. Reproduction rate

We employed a 2 × 2 repeated measures factorial design to investigate the effects of two main factors, rearing temperature and sodium alginate concentration, on the infectivity of EPNs. The experiment was conducted at three time points (1, 100, and 180 days post-formulation) at which the final instar larvae of *T. molitor* were infected with IJs encapsulated in calcium alginate beads. Each experimental unit consisted of 10 last instar larvae of *T. molitor*, transferred to 8 cm diameter Petri dishes, and co-inoculated with 35 IJs of each nematode species. Duplicate Petri dishes were used for each treatment combination. The dead larvae were collected and retained in the White trap until emergence of IJs from the cadavers. Daily counts were conducted until no further nematodes were recovered from the cadavers. This entire experiment was replicated twice.

### 2.7. Analysis data

The survival rate of formulated IJs was analyzed using a two-way analysis of variance (ANOVA) in SAS/STAT (SAS Institute, 1989). A slicing approach was employed to assess significant differences between means. Two main effects were considered: sodium alginate concentration (1.5% and 1.75%) and time interval post-formulation (1, 10, 20, 30, 40, 50, 60, 70, 80, 90, 100, 120, 150, and 180 days). The effects of each factor alone and their interactions were examined, with data analyzed separately for each storage temperature. Following significant ANOVAs (*p* < 0.05), least significant difference (LSD) tests were conducted in SAS software to determine differences between means within each factor. All values in the figures are presented as mean ± standard error (SE).

In a first step, we employed a two-way ANOVA in SAS/STAT (SAS Institute, 1989) to investigate the reproduction rate of formulated IJs. The main effects of two independent variables, days post-formulation (1, 100, and 180 days) and rearing temperature (8°C and 25°C), were examined. In addition, the interaction between these two factors, namely the day post-formulation × rearing temperature interaction, was assessed. To identify significant differences between means, a slicing approach was employed. In a second step, the main effects of sodium alginate concentration (1.5% and 1.75%) and time intervals post-formulation (1, 100, and 180 days) were investigated. The effects of each factor alone and their interaction were examined separately for each storage temperature (8°C and 25°C). Statistical significance (*p* < 0.05) in the ANOVAs prompted further analysis of differences between means within each factor using the LSD test in SAS software. Mean values in the figures are presented as mean ± SE.

## 3. Results

### 3.1. Formulation

#### 3.1.1. Optimizing ingredient concentrations

*Sodium alginate*: The physical characteristics of the beads varied significantly with sodium alginate concentration. Beads prepared at 0.5% and 1% were found to be soft and prone to disintegration upon gentle pressure. Conversely, the beads formed at a 2% concentration demonstrated a more intricate morphology, although their shapes were irregular. In contrast, beads formulated at 1.5% and 1.75% concentrations exhibited a complex and circular geometry, with diameters of approximately 4 mm. These latter concentrations were subsequently identified as optimal for bead formation.

*CaCl2*: The impact of CaCl_2_ concentration on the physical characteristics and stability of beads formulated for encapsulating nematodes was investigated. Beads prepared in 10 mM and 15 mM CaCl_2_ were found to be soft and exhibit irregular shapes, whereas those formed in 20 mM and 30 mM CaCl_2_ had a more complex and regular morphology, which was resistant to mechanical disruption. Notably, the IJs encapsulated in the latter beads escaped within 24 hours after transfer to water. In contrast, beads formulated in 50 mM and 100 mM CaCl_2_ were found to be robust, effectively preventing IJs’ escape even after water transfer. Based on these observations, 20 mM and 30 mM CaCl_2_ concentrations were selected for subsequent experiments. However, a subsequent evaluation revealed that the 20 mM concentration was unsuitable for long-term formulation storage due to the escape of IJs from the beads stored at 25°C and 8°C within a month, even in the absence of water transfer.

*CaCl2 : glycerin ratio:* The formulation with an 8:2 ratio of CaCl_2_ to glycerin was selected as the most suitable for encapsulation purposes. This was determined by its demonstrated physical strength, regular shape, and ability to prevent premature IJ nematode escape, thereby ensuring the integrity of the encapsulated formulation.

#### 3.1.2. Physical characteristics of formulation

**Figure 2:**
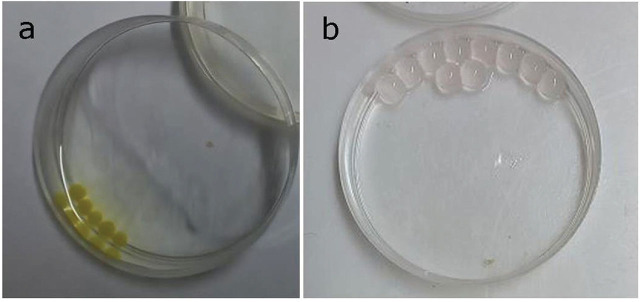
Calcium alginate beads: A) At the time of transfer to water and B) 24 h after transfer to water.

One ml of the formulation suspension yielded 90 beads, each containing approximately 150 IJs. Ten calcium alginate beads placed into 4 ml of sterile water absorbed approximately 400 μl of water over a period of 24 hours, doubling in volume. This swelling created gaps in the beads, facilitating the emergence of the IJs (Fig. 2). We identified two compounds that acted as absorbents when added to the sodium alginate suspension, given that beads formulated without these compounds did not exhibit swelling. The two absorbent compounds are pending patent protection.

#### 3.1.3. Formulation efficacy

The effectiveness of the formulation with respect to nematode longevity and pathogenicity was quantified over a period of 180 days following encapsulation of IJ nematodes within beans at either 8ºC or 25ºC.

*Heterorhabditis bacteriophora:* The main effects of sodium alginate concentration (F_2,90_ = 13130.7, *p* <.0001, F_2,90_ = 1693.31, *p* <.0001) and aging duration of the formulation (F_14,90_ = 1872.86, *p* <.0001, F_14,90_ = 791.74, *p* <.0001) significantly affected the survival of *H. bacteriophora* at both 25ºC and 8ºC, respectively (Table 1). There was a significant interaction of alginate concentration and aging duration of the formulation at both 25ºC (F_28,90_ = 347.20, *p* <.0001) and 8ºC (F_28,90_ = 331.38, *p* <.0001) (Table 1).

**Table 1:** The survival rate of *Heterorhabditis bacteriophora* IJs escaping from calcium alginate beads. The control treatment was kept in sterile water.

**Temperature**	**Treatments**	**1-day**	**10-day**	**20-day**	**30-day**	**40-day**	**50-day**	**60-day**	**70-day**	**80-day**	**90-day**	**100-day**	**110-day**	**120-day**	**150-day**	**180-day**
25ºC	SA 1.5%	100a	99.34a	82.95d	71.76e	71.45e	70.91ef	69.64f	68.94f	68.67f	68.52f	68.29fg	67.52g	67.52g	61.73h	59.8h
	SA 1.75%	84.49cd	83.53d	82.68d	82.33d	70.99e	66.94g	63.97g	51.89i	50.27i	49.58i	48.8ij	47.61i	46.45jk	44.9k	41.37k
	Control	100a	94.33b	86c	54hi	24.67i	8.67m	0n	0n	0n	0n	0n	0n	0n	0n	0n

8ºC	SA 1.5%	47.49g	35.69ij	35.19j	34.84j	34.53j	33.37j	31.75jk	31.02k	29.51k	28.24k	27.08kl	26.54kl	25.23l	23.61l	23.26l
	SA 1.75%	63.46e	62.11ef	49.19g	47.61g	46.72g	46.03g	44.95g	44.48g	43.67gh	42.48h	38.39i	38.27i	38.39i	37.96i	37.81i
	Control	100a	100a	100a	100a	86.67b	78c	69d	58f	32.67n	26.33kl	21.67l	16m	14m	0n	0n

SA: Sodium alginate.

Numbers are expressed as a percentage. Means between lines followed by the same letter are not significantly different at the *P* = 0.05 LSD test.

The most effective alginate concentration and temperature for long-term storage of *H. bacteriophora* in beads were 1.5% and 25ºC, respectively. The survival rate of stored IJs at 25ºC was 59.8% at 180 days post formulating. At 8ºC, the survival rate decreased to 23.6% (Table 1). Survival of IJs was lowest in the water control treatment, with only 8% of IJs remaining alive at 50 days post-initiation of the experiment.

*Steinernema carpocapsae:* The main effects of sodium alginate concentration (F_2,90_ = 5592.81, *p*<.0001, F_2,90_ = 2030.71, *p* <.0001) and aging duration of the formulation (F_14,90_ = 956.36, *p* <.0001, F_14,90_ = 1028.55, *p* <.0001) significantly affected survival of *H. bacteriophora* at both 25ºC and 8ºC, respectively (Table 2). There was a significant interaction of alginate concentration and aging duration of the formulation at both 25ºC (F_28,90_ = 161.50, *p* <.0001) and 8ºC (F_28,90_ = 249.03, *p* <.0001) (Table 2).

**Table 2:** The survival rate of *Steinernema carpocapsae* IJs escaping from calcium alginate beads.

**Temperature**	**Treatments**	**1-day**	**10-day**	**20-day**	**30-day**	**40-day**	**50-day**	**60-day**	**70-day**	**80-day**	**90-day**	**100-day**	**110-day**	**120-day**	**150-day**	**180-day**
25ºC	SA 1.5%	85.11b	81.91c	75.8de	72.13e	70.27e	68.56f	63.62g	57.82hi	57.23i	56.06i	54.95ij	53.83j	52.61j	41.81l	26.6n
	SA 1.75%	70.43e	65.16g	64.47g	63.09gh	61.49h	59.57h	53.72j	52.39j	50.85jk	50.16k	48.51k	47.55k	46.81k	37.23m	10.64p
	Control	100a	78.33d	45kl	43.33l	35m	14.33o	5q	0r	0r	0r	0r	0r	0r	0r	0r

8ºC	SA 1.5%	100a	90.96c	83.4de	76.6ef	70.53g	68.56gh	67.29gh	66.65h	65.53h	59.31i	57.45j	56.06jk	55.32jk	54.41jk	47.87m
	SA 1.75%	82.45de	80.32e	79.36e	78.03ef	76.28f	75.11f	73.67fg	72.61fg	70.96g	69.79gh	68.35gh	67.29gh	60.9i	53.83k	50.16l
	Control	100a	97.33ab	94.67b	91c	86d	71.33g	55.67jk	43.67n	31o	18.33p	10.33q	2.67r	0s	0s	0s

SA: Sodium alginate.

The control treatment was kept in sterile water. Numbers are expressed as a percentage. Means between lines followed by the same letter are not significantly different at the *P* = 0.05 LSD test.

Survival of *S. carpocapsae* within alginate beads was higher at 8ºC than that at 25ºC; However, IJs exhibited higher survival in alginate beads than in water (negative control) at 25ºC (Table 2). By 50 days of formulation aging, only 14.33% of IJs were alive in water control, while survival within formulated beads was still greater than 50% (Table 2).

*Steinernema feltiae:* The main effects of sodium alginate concentration (F_2,90_ = 1010.11, *p* <.0001, F_2,90_ = 494.93, *p* <.0001) and aging duration of the formulation (F_14,90_ = 1732.38, *p* <.0001, F_14,90_ = 3613.13, *p* <.0001) significantly affected survival of *H. bacteriophora* at both 25ºC and 8ºC, respectively (Table 3). There was a significant interaction of alginate concentration and aging duration of the formulation at both 25ºC (F_28,90_ = 222.65, *p* <.0001) and 8ºC (F_28,90_ = 169.82, *p* <.0001) (Table 3).

**Table 3:** The survival rate of *Steinernema feltiae* IJs escaping from calcium alginate beads.

**Temperature**	**Treatments**	**1-day**	**10-day**	**20-day**	**30-day**	**40-day**	**50-day**	**60-day**	**70-day**	**80-day**	**90-day**	**100-day**	**110-day**	**120-day**	**150-day**	**180-day**
25	SA 1.5%	71.43d	67.05e	61.81g	59.14g	53.33hi	50.38i	43.14j	40.1jk	38.57jk	37.38jk	31.9l	19.76o	15.14p	0r	0r
	SA 1.75%	49.52i	62.1fg	58.76g	55.05h	50.76i	44.29j	41.9jk	40.86jk	35.71k	32.33l	30.1l	28.05mn	25.86n	0r	0r
	Control	100a	88b	75.67c	51.67i	28.67m	14.33p	4.33q	0r	0r	0r	0r	0r	0r	0r	0r

8	SA 1.5%	100a	76.67f	66.67h	53.43j	44.48kl	46.67k	34.29n	31.9no	27p	20.86qr	19.67r	14.29s	15.48s	0v	0v
	SA 1.75%	93.33cd	83.52e	62.86i	53.33j	46.67k	46.43k	39.38m	34.52n	32.67n	32.62no	30.1o	26.52p	22.86q	0v	0v
	Control	100a	87.33b	84.33c	79.67d	70.67e	62.67g	50.33lj	38.67o	28o	19.33r	16t	10.33u	4.67v	0v	0v

SA: Sodium alginate.

The control treatment was kept in sterile water. Numbers are expressed as a percentage. Means between lines followed by the same letter are not significantly different at the *P* = 0.05 LSD test.

The survival of *S. feltiae* IJs formulated within alginate beads was similar at both temperatures. However, the survival of *S. feltiae* IJs in the control treatment (water) was lower at 25ºC than 8ºC (Table 3).

### 3.2. Pathogenicity test

The stability of pathogenicity in formulated EPNs was investigated over a 6-month period. The lethality rate of IJs remained at 100% at both 25°C and 8°C storage temperatures. In contrast, the pathogenicity rate of negative control treatments decreased significantly over time compared to the formulated nematodes. For *H. bacteriophora* at 25°C, the pathogenicity rate decreased from 100% on the first day to 30% on the 50th day. Similarly, at 8°C, the mortality rate decreased from 100% on the first day to 20% on the 120th day. For *S. carpocapsae* at 25°C, the pathogenicity rate decreased from 100% on the first day to 40% on the 50th day. At 8°C, the mortality rate decreased from 100% on the first day to 20% on the 110th day. For *S. feltiae* at 25°C, the pathogenicity rate decreased from 100% on the first day to 10% on the 60th day. Similarly, at 8°C, the pathogenicity rate decreased from 100% on the first day to 10% on the 120th day. These results indicate that the formulated EPNs retained their pathogenicity over a 6-month period, while the pathogenicity of the negative control treatments decreased significantly over time.

### 3.3. Reproduction of formulated nematodes

#### 3.3.1. Heterorhabditis bacteriophora

**Figure 3:**
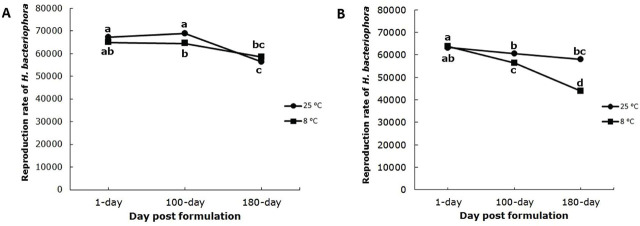
Reproduction rate of *Heterorhabditis bacteriophora* in last instar larvae of *Tenebrio molitor* at two storage temperatures, 8ºC and 25ºC and after 1, 100, and 180 days of formulation aging. A) Formulated with 1.5% sodium alginate, B) formulated with 1.75% sodium alginate.

There was no significant (F_2,36_=2.36, *p* = 0.1089) interaction of aging duration and storage temperature (8ºC and 25ºC) on the reproduction of nematodes in beads formulated with 1.5% sodium alginate. Aging duration significantly (F_2,36_=23.21, *p* = <0.0001) affected nematode reproduction, but storage temperature did not (F_1,36_=1.54, p=0.2232) (Fig. 3A).

The interaction between aging duration and storage temperature significantly (F_2,36_=7.19, *p* = 0.0024) affected nematode reproduction in beads formulated with 1.75% sodium alginate. The main effects of aging duration (F_2,36_=29.24, *p* = <0.0001) and storage temperature (F_1,36_=29.79, *p* = <0.0001) both significantly affected nematode reproduction (Fig. 3B).

The reproduction of *H. bacteriophora* decreased over time in both formulations of alginate beads and was generally higher at 25ºC than 8ºC. There was no significant difference in nematode reproduction between the two concentrations of sodium alginate at 25ºC (F_1,36_=1.54, *p* = 0.22), but reproduction was significant greater at the 1.5 than 1.75% concentration of sodium alginate at 8ºC (F_1,36_=29.79, *p* = <0.0001).

#### 3.3.2. Steinernema carpocapsae

**Figure 4:**
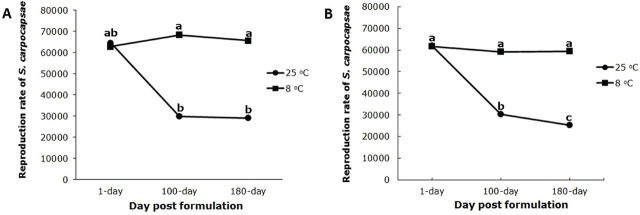
Reproduction rate of *Steinernema carpocapsae* in the last instar larvae of *Tenebrio molitor* at two storage temperatures, 8ºC and 25ºC and after 1, 100, and 180 days of formulation aging. A) formulated with 1.5% sodium alginate, B) formulated with 1.75% sodium alginate.

The interaction between aging duration and storage temperature significantly (F_2,36_=124.46, *p* < 0.0001) affected nematode reproduction in beads formulated with 1.5% sodium alginate. Both aging duration (F_2,36_=74.90, *p* < 0.0001) and storage temperature (F_1,36_=416.91, *p* < 0.0001) significantly affected nematode reproduction (Fig. 4A).

The interaction between aging duration and storage temperature significantly (F_2,36_=88.03, *p* < 0.0001) affected nematode reproduction in beads formulated with 1.75% sodium alginate. Both aging duration (F_2,36_=115.16, *p* < 0.0001) and storage temperature (F_1,36_=335.76, *p* < 0.0001) significantly affected the reproduction of *S. carpocapsae* (Fig. 4B).

Reproduction of *S. carpocapsae* did not differ between the two alginate concentrations, but reproduction decreased over time. Reproduction was higher at 8ºC than 25ºC with both concentrations of sodium alginate, but there was no significant difference between the two concentrations of sodium alginate at 25ºC (F_1,36_=3.12, *p* = 0.085). However, at 8ºC, reproduction of *S. carpocapsae* was significantly higher at the 1.5 than 1.75% concentration of sodium alginate (F_1,36_=21.47, *p* < 0.0001). Nematode reproduction decreased by almost 50% in both alginate bead formulations at 25ºC, while storage at 8ºC had a negligible effect on reproduction.

#### 3.3.3. Steinernema feltiae

**Figure 5:**
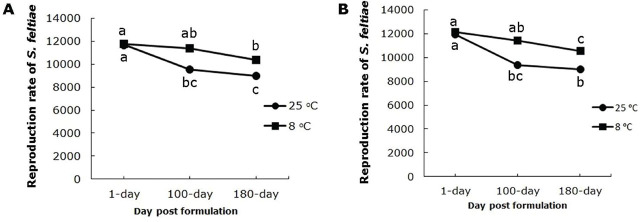
Reproduction rate of *Steinernema feltiae* in the last instar larvae of *Tenebrio molitor* at two storage temperatures, 8ºC and 25ºC and after 1, 100, and 180 days of formulation aging. A) formulated with 1.5% sodium alginate, B) formulated with 1.75% sodium alginate.

There was no significant (F_2,36_=2.81, *p* = 0.0.073) interaction between aging duration and storage temperature on reproduction of nematodes in beads formulated with 1.5% sodium alginate. The main effects of aging duration (F_2,36_=14.01, *p* < 0.0001) and storage temperature (F_1,36_=12.03, p=0.0014) both significantly affected the reproduction of *S. feltiae* (Fig. 5A).

There was no significant (F_2,36_=2.58, p=0.089) interaction effect between aging duration and storage temperature on reproduction of *S. feltiae* in beads formulated with 1.75% sodium alginate. Both aging duration (F_2,36_=16.14, *p* < 0.0001) and storage temperature (F_1,36_=13.51, *p* = 0.0008) significantly affected nematode reproduction (Figure 5B). Reproduction of *S. feltiae* decreased over time and was similar with both concentrations of sodium alginate at each storage temperature evaluated (F_1,36_=0.34, *p* = 0.56 (Fig. 5B).

## 4. Discussion

EPNs can be formulated in various ways to promote long-term storage and transportation to the field as biopesticides (Nxitywa and Malan, 2021). Ehlers (2007) suggested that the formulation of nematode IJs could help reduce nematode mortality after emergence. Furthermore, the physical attributes of a formulation and abiotic conditions during storage, such as temperature, can be controlled to facilitate the duration of storage and successful delivery to the field (Grzywacz et al., 2014; Ruiz-Vega et al., 2018).

Sponges are commonly used to store and transport EPN IJs in small volumes. However, sponges are not an optimal substrate for maintaining high densities of IJs because the nematodes are capable of escaping, given their mobility. Excessive nematode movement causes depletion of energy and desiccation (Grewal and Peters, 2005). Additionally, use of sponges is costly and requires refrigeration during storage and transportation (Grewal, 2002; Cruz-Martínez et al., 2017). Andalo et al. (2011) reported that the survival rate of *S. carpocapsae* in sponge formulations at 16°C was 89.3% after 90 days and 57.5% at 180 days post-formulation. Similarly, Grewal (2002) reported survival durations of EPNs between 1–3 months in sponges at 5ºC −10°C. A further disadvantage of using sponges is the complexity of extracting and isolating IJs from the sponge matrix, which can hinder downstream applications.

In order to be effective, an ideal formulation should possess high quality and efficacy. Moreover, it should be easily transportable and applicable in field settings. Recent research has focused on developing IJ formulations that can be stored at high concentrations for extended periods without a decline in pathogenicity (Shapiro-Ilan et al., 2012). Various formulations have been developed, including wettable powders, water-dispersible granules, surfactants, oil emulsions, and alginate beads (Grewal, 2002; Beck et al., 2013; Noosidum et al., 2016; Aquino et al., 2019; Platt et al., 2019; Fallet et al., 2024; Nxitywa and Malan, 2021). Alginate formulations are particularly noteworthy due to their environmentally benign nature, making them suitable for field use (Nxitywa and Malan, 2021). Furthermore, their use to formulate EPNs is cost-effective and they are easy to apply (Vemmer and Patel, 2013).

Herein, we investigated an alginate bead formation using various concentrations of sodium alginate and CaCl_2_. The escape rate, longevity, and pathogenicity of IJs were monitored over time. Our findings indicate that the escape rate of IJs from alginate beads can be reduced for extended periods by manipulating the formulation to circumvent the challenges of long-term storage (Kagimu and Malan, 2019). IJs were observed escaping from softer formulations of alginate beads, consistent with the results of Hiltpold et al. (2012). Specifically, IJs escaped prematurely from beads produced with 0.5% or 1% sodium alginate and 10, 15, or 20 mM CaCl_2_ prior to transfer into water. Conversely, beads produced with 1.5% and 2% sodium alginate retained IJs more effectively, with the nematodes escaping only upon transfer to water. This outcome is in agreement with previous findings by Hiltpold et al. (2012). The concentration of alginate has a direct impact on bead hardness, with increasing alginate concentration resulting in harder beads. As bead hardness increases, the number of nematodes escaping after transfer to water decreases. Furthermore, beads formed with 2% sodium alginate exhibited an irregular shape, which hindered IJ escape.

Calcium chloride concentration also had an effect on bead hardness, consistent with previous findings (Kim et al., 2015). Beads formed in 10 mM CaCl_2_ and 15 mM CaCl_2_ solutions were fragile and exhibited irregular morphologies, readily deforming under gentle pressure. Conversely, beads formed at higher concentrations of CaCl_2_ (20 and 30 mM) demonstrated increased structural complexity and regular shapes, resisting deformation under equivalent pressure. Notably, IJs encapsulated in these beads exhibited rapid escape upon transfer to water, with all individuals escaping within 24 hours. In contrast, beads formed at higher concentrations of CaCl_2_ (50 and 100 mM) were tougher and resistant to IJ escape, with no individuals escaping within the same time frame. These findings suggest that a CaCl_2_ concentration of 30 mM is optimal for the current formulation. In contrast to our results, Nxitywa and Malan (2021) found that while varying alginate concentration significantly affected the number of IJs escaping from beads, Ca^2+^ concentration and hardening time did not yield significant differences. For the currently described formulation, beads were prepared with 1.5–1.75% sodium alginate and 50–100 mM CaCl_2_, and allowed 1-minute to harden, effectively retaining IJs until immersion in water.

Previous studies have demonstrated that the storage temperature of encapsulated EPNs significantly affects their viability and reproductive potential. Kagimu and Malan (2019) reported that beads stored at 25°C retained a greater number of IJs than those stored at lower temperatures (8°C and 14°C). Our results show that EPNs stored at 25°C can maintain viability for up to 180 days, exceeding the longevity observed at 8°C. This suggests that refrigeration may not be necessary for the storage of EPNs, potentially reducing storage-related costs. The reproductive potential of *H. bacteriophora* was lower at 8°C compared to 25°C, while the opposite trend was observed with *S. carpocapsae* and *S. feltiae*. This is in agreement with previous studies, which have reported varying survival rates for different EPN species at different storage temperatures (Ruiz-Vega et al., 2018; Kary et al., 2018; Hussein and Abdel-Aty, 2012). For example, Ruiz-Vega et al. (2018) reported survival times of 35, 28, and 14 days for *H. bacteriophora*, *S. carpocapsae*, and *S. feltiae*, respectively, formulated in alginate beads stored at room temperature (25°C). Similarly, Kary et al. (2018) found that glycerol-dehydrated *H. bacteriophora* stored in sodium alginate beads had a 40% survival rate after 60 days, which decreased to 10% after 90 days. In contrast, Nxitywa and Malan (2023) reported high survival rates for IJs of *Steinernema yirgalemense* stored in agar formulations at 25°C, with 77% and 82% survival rates recorded after 6 weeks for 1% and 1.5% agar formulations, respectively.

Temperature is a critical factor affecting the survival of IJs (Stuart et al., 2015; Kagimu et al., 2017). The optimal storage temperature for EPNs is a topic of ongoing debate. According to Poinar (1979), *Steinernema* species are cold-tolerant, with an optimal storage temperature of 5-9°C, while the preferred storage temperature for *Heterorhabditis* species is 12°C (Bedding, 1981). However, our findings suggest that EPNs can be stored at 25°C for up to 180 days without significant loss of viability. In the current study, we surveyed the survival rate of EPNs for up to 6 months at 25°C. The survival rate for *H. bacteriophora* and *S. carpocapsae* was 60% and 50% within 3 months after formulation, respectively, while *S. feltiae* had a 30% survival rate in the third month. Understanding the effects of temperature on the survival and reproductive potential of EPNs will be essential for optimizing storage conditions and improving the efficacious use of these beneficial organisms in biological control programs.

Our study demonstrates that beads formulated with 1.5% sodium alginate are a suitable matrix for storing EPNs at room temperature for extended periods. Notably, at least 50% of the nematodes remained viable and capable of escaping from beads within 24 hours after transfer to water, even after three months of storage. The dissolution of calcium alginate beads in water facilitates the escape of EPNs from the bead matrix. Upon transfer to water, the beads absorb water, soften, swell, and cleave, resulting in the formation of slits that allow the nematodes to escape. This characteristic makes calcium alginate beads a versatile formulation for both aquatic and terrestrial applications. The applicability of calcium alginate beads to soil has also been explored in preliminary tests in which EPNs escaped from beads after transfer to soil followed by irrigation. This suggests that these beads could be applied to soil during seed planting, offering a novel approach for the delivery of beneficial nematodes to agricultural fields. Our results provide a foundation for further research on optimization of bead alginate formulations. Future work is needed to investigate the effects of varying sodium alginate concentrations, bead sizes, and hardening times on the survival rate and infectivity of EPNs. Additionally, more replication across a larger number of EPN species is necessary to ensure generalizability of the current results.

In this research, our hypothesis that nematodes survive longer in the formulation than in sterile water was confirmed. We identified two compounds that acted as absorbents when added to the sodium alginate suspension. These compounds absorb water and cause the beads to swell, creating gaps in the beads that facilitate the emergence of the IJs. The results showed that alginate beads were able to maintain EPNs at room temperature for three months without reducing their pathogenicity. Therefore, there is no need to use a refrigerator to transport nematodes to the field or greenhouse. The survival of nematodes at room temperature and maintaining their ability to be pathogenic within a formulation is important and this was achieved with this formulation. However, this formulation also has limitations.

Large-scale production of alginate beads is currently limited, and further research is also needed on mass production methods to meet the demand for these beneficial organisms. Research efforts should focus on increasing the survival of IJs over time at room temperature and optimizing the number of IJs per bead to maximize their efficacy in biological control programs. In line with previous studies (Ruiz-Vega et al., 2018), the survival rate and infectivity of EPNs may be related to the fitness of each EPN species tested. Therefore, further research is also needed to understand the factors influencing the survival and infectivity of EPNs deployed using beads within different environments.
